# A Case of Undifferentiated Carcinoma in a 2-Month Postpartum Reproductive Tract with a Rapid and Fulminant Course

**DOI:** 10.1155/2021/3516646

**Published:** 2021-10-19

**Authors:** Yasuhiro Yokoyama, Mariko Suzuki, Yasumasa Sato, Shegenori Iwagaki, Yuichiro Takahashi, Hitoshi Iwata

**Affiliations:** ^1^Department of Obstetrics & Gynecology, Gifu Prefectural General Medical Center, 4-6-1 Noishiki, Gifu, Gifu 500-8717, Japan; ^2^Department of Pathology, Gifu Prefectural General Medical Center, 4-6-1 Noishiki, Gifu, Gifu 500-8717, Japan

## Abstract

Advanced carcinoma of the lower female reproductive tract is rare during pregnancy and the postpartum period. We here present a case of a 32-year-old Japanese woman, whose entire lower reproductive tract had been invaded by carcinomas as of 2 months after childbirth. She had been infertile, and pregnancy had been established by repeated embryo transfer. The gynecological cancer screening, which included Pap smear tests, was negative during the periods she underwent infertility treatment or during the first trimester. At 26 gestational weeks, the patient noticed uterine contractions concomitant with genital bleeding. Labor progressed slowly and steadily; thus, the pregnancy was ended by cesarean section at 29 weeks. At 2 months after childbirth, the patient experienced increased left abdominal pain and underwent a pelvic examination, revealing multiple pelvic masses and diffuse vaginal tumors causing stenosis. Vaginal tumors were biopsied, and histochemical analysis showed undifferentiated carcinoma with possible adenocarcinoma. Imaging modalities including CT, MRI, and PET-CT suggest that the carcinoma had invaded the entire reproductive tract, especially the uterine body, metastasized into the lungs and the ischial bones, and disseminated onto the peritoneum. She received multiple rounds of chemotherapy but died 6 months after childbirth. Taking into consideration the clinical feature and immunohistochemical profiles of the cancer cells, the endometrium is the most likely origin.

## 1. Introduction

Carcinomas of the reproductive systems of pregnant and postpartum women are rare. However, of these carcinomas, invasive cervical cancer of the uterine cervix is the most common, with an incidence of 1.2 per 10,000 pregnancies [1], followed by ovarian carcinomas, with an incidence of 4-8 per 100,000 pregnancies [2]. Endometrial carcinomas are extremely rare during pregnancy and the postpartum period; a limited number of cases have been reported in previous works [3–6]. In most cases, the pregnancies ended in spontaneous or induced abortion in an early gestational stage.

We here report a patient who was diagnosed with advanced carcinoma of the female reproductive tract at 2 months postpartum. The pregnancy resulted in premature delivery at 29 gestational weeks. Two months after the childbirth, cancer was detected across much of the patient's reproductive tract. Its primary site may have been the uterine corpus.

## 2. Case Report

A 32-year-old Japanese women, gravida 0, para 0, had been infertile for 3 years. The cause of the infertility had been investigated extensively, but no clear reason was found. Pap smear tests for cervical and endometrial cancers performed at 6 months before the pregnancy were negative. Pregnancy was established by repeated embryonal transfer.

Irregular vaginal bleeding was sometimes noted even during the first trimester. A Pap smear test performed at 10 gestational weeks was negative for intraepithelial lesions or malignancy (NILM). The patient was hospitalized at 26 gestational weeks due to increasing uterine contractions, genital bleeding, and shortening of the uterine cervix (2.3 cm). Magnesium sulfate and ritodrine chloride were administered, but the disease progressed rapidly. Because of the transverse presentation of the baby and a bulge in the membrane, a cesarean section was performed at 29 gestational weeks. The baby weighed 1124 g with an Apgar score of 4/6. Pathological examination of the placenta showed grade 3 chorioamnionitis.

**Figure 1:**
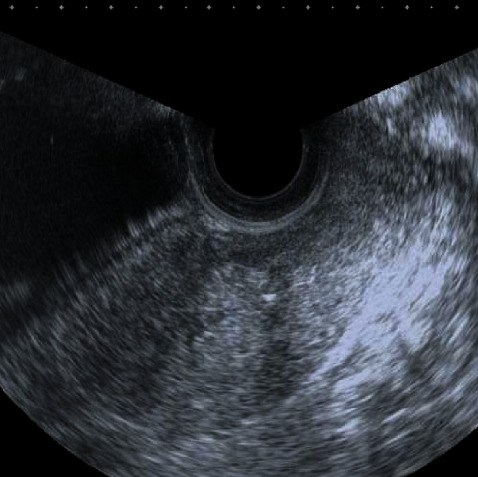
Ultrasound view of the uterus 1 month after childbirth. The longitudinal length of the uterus is 8.2 cm. No abnormal structures are visible in either the uterine body or the cervix.

The patient was discharged from the hospital on the7^th^ day after the surgery. At that time, ultrasound showed that the uterus had shrunk to 12-week size and the endometrium was thin. An obstetrical check performed one month postpartum indicated that the uterus was atrophic, and no unusual findings were detected upon pelvic ultrasound (Figure 1).

At 2 months after childbirth, the left abdominal had worsened, so the patient visited us. A pelvic examination revealed multiple pelvic masses and a diffuse vaginal tumor causing stenosis. An ultrasound scan of the pelvis revealed a slightly hyperechogenic uterus of 6-week size, with multiple peritoneal nodules and ascites, while the endometrium was still thin. Pap smear of the uterine cervix showed NILM again.

**Figure 2:**
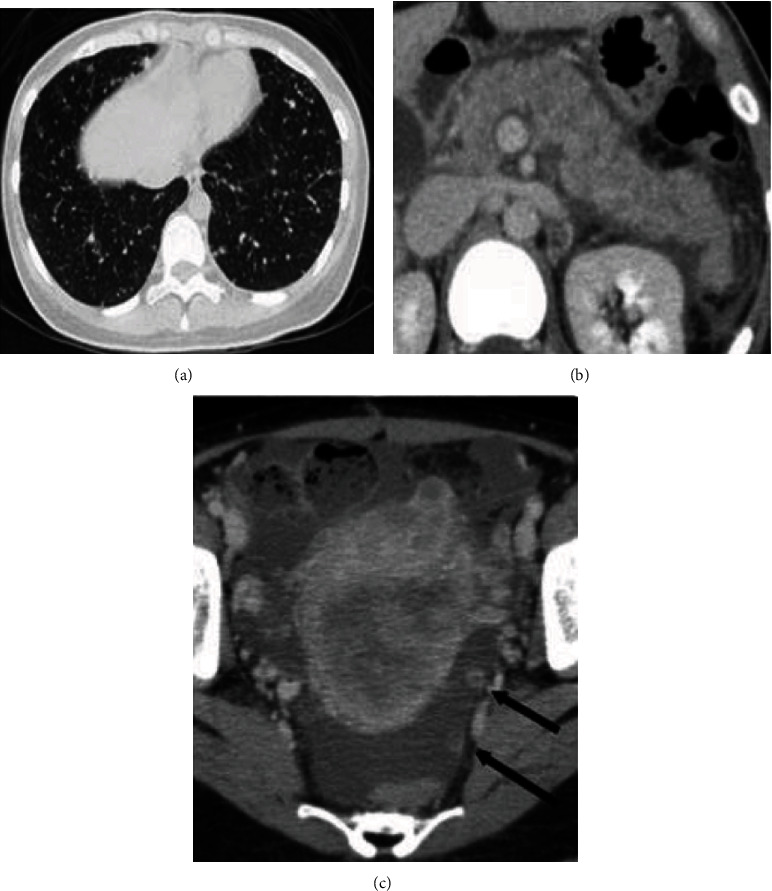
Contrast-enhanced computed tomography. (a) Numerous nodules were visible in bilateral lungs. (b) The pancreas is edematous and surrounded by effusion. (c) The uterus was enlarged, and the myometrium was irregularly stained. On the pelvic peritoneum, nodules were found (arrows).

A CT scan showed multiple nodules in the lungs and on the peritoneum. The uterus was enlarged and irregularly stained in contrast-enhanced CT (Figure 2). The pancreas was diffusely swollen with small amount of effusion surrounding it.

**Figure 3:**
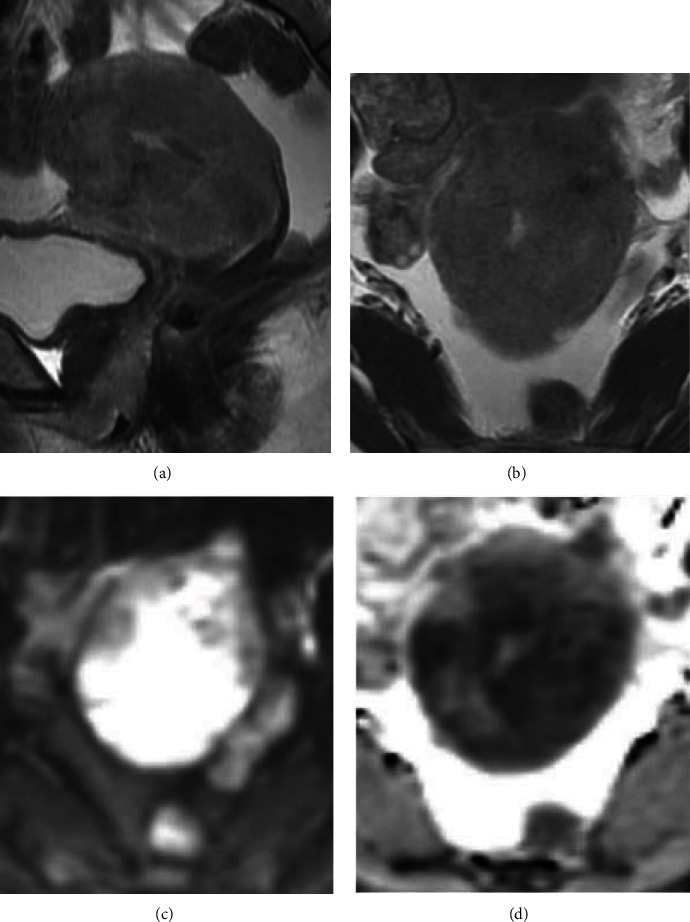
(a) Sagittal T2-weighted MRI image of the uterus and the vagina: the myometrium signal intensity was higher than that of the intestinal stroma. Similar intensity was observed in the lower vagina, the vulva, and a nodule on Douglas' pouch. (b) Axial T2-weighted MRI image of the uterus and the pelvis. The signal intensity of the myometrium was higher than that of the coccygeus muscle. (c) Axial diffusion-weighed image of the uterine body. The entire myometrium showed high intensity. (d) Apparent diffusion coefficient (ADC) map. The mean ADC value of the whole uterus was 0.950 × 10^−3^ mm^2^/s with 0.720 × 10^−3^ mm^2^/s of it being covered by the darkest shade.

T2-weighted MRI revealed that the endometrium was atrophic, but the myometrium was hypertrophic and the junctional zone had disappeared. The uterine cervix appeared normal. The vaginal thickness was observed in its lower segment. Diffusion-weighted MRI of the uterus and the vagina suggested that the entire uterus might have been replaced by malignant tissue, which had reached deep into the lower segment of the vagina (Figure 3).

**Figure 4:**
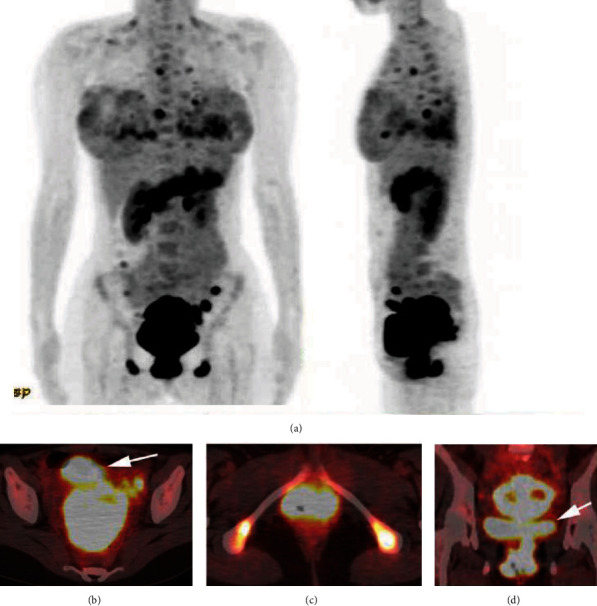
(a) FDG-PET/CT images of the whole body. Abnormal uptake of FDG was observed through the whole body, although it was strongest in the reproductive tract. (b) Axial FDG-PET/CT images of the uterine body. Urine (arrow) obscured FDG accumulation in the reproductive tract, whereas the uterus and the left ovary showed clear uptake of FDG. (c) Axial image of the ischial tuberosities and the vulva. (d) Coronal image of the uterus and the vagina. Excreted FDG into the bladder (arrow) was overlaid on the uptake in the reproductive tract.

In PET-CT, abnormal accumulations of fluorine 18 fluorodeoxyglucose (FDG) were observed throughout the uterus, vagina, and pancreas and parts of the vulva, ovary, bilateral ischial tuberosities, peritoneal nodules, lungs, and mediastinal and pelvic lymph nodes (Figure 4).

**Figure 5:**
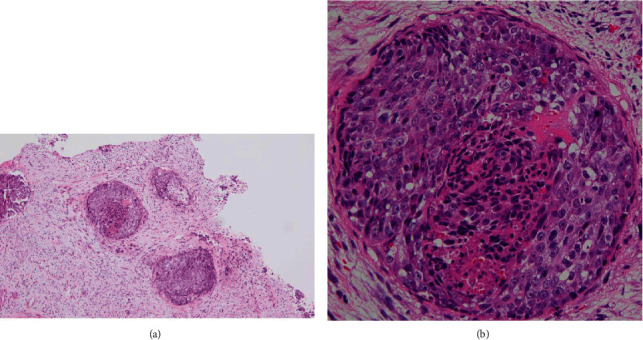
(a) Low-power view of the vaginal tumor. Malignant cells infiltrated into submucosal tissues, forming tumor nests. (b) High-power view. Undifferentiated cells surrounded a necrotic cluster.

Vaginal tumors were biopsied and subjected to histological examination via hematoxylin and eosin staining. Results showed undifferentiated carcinoma (Figure 5). Tumors were further investigated by immunostains and fluorescence in situ hybridizations. Percentages of cell staining were recorded: negative (0-5%), 1+ (6-25%), 2+ (26-50%), 3+ (51-75%), and 4+ (>75%). The staining results are summarized in Table 1. Despite these results, however, the origin of the cancers remained uncertain. The pathological diagnosis was finally determined as undifferentiated carcinoma of unknown origin with possible adenocarcinoma.

Serum levels of CA125, CA199, and CEA reached 324.8 IU/ml, 123.2 IU/ml, and 6.0 mg/dl, respectively. Given the well-preserved structure of the uterine cervix, repeated negative cytology from the uterine cervix, and irregularly enlarged uterine body, we finally diagnosed the patient with an endometrial carcinoma of FIGO stage 4b.

Regarding the abnormal FDG accumulation in the pancreas, hypercalcemia-induced edematous pancreatitis was conceived. Urinastatin administration rapidly diminished serum amylase and lipase, and the pancreatitis was completely cured within a month.

Chemotherapy with paclitaxel and carboplatin was repeatedly performed, but the patient died 6 months after childbirth.

## 3. Discussion

To identify the primary site of undifferentiated carcinoma of unknown origin, clinical findings, radiological images, and histopathological profiles of neoplastic cells must be taken into full consideration. In this case, radiological images appear to indicate an origin in the reproductive tract. An immunohistochemical approach failed to specify its origination, and only with great scrutiny could we determine that it was an adenocarcinoma; from immunohistochemical results alone.

In the lower female reproductive tract, adenocarcinomas mostly arise from the uterine cervical glands or the endometrium. Endometrial and cervical glands are morphologically different, so these cancers can be distinguished from each other from their locations and morphological features in most cases. The discrimination process, however, is sometimes difficult, especially in the cases of the small specimens available for biopsy and those of high-grade tumors at the isthmus of the uterus. Immunohistochemical staining can help identify these cancers. Estrogen receptors and vimentin are preferentially expressed in endometrial carcinomas, whereas CEA is expressed in the endocervical adenocarcinoma [7–9]. p16 is a surrogate marker of HPV-related lesions of the uterine cervix, but histochemical studies have shown that it is expressed in most endometrial carcinomas [10–12]. In this case, the immunohistochemical profile did not specify the primary site of the tumor within the uterus.

Undifferentiated carcinoma of the uterus is rare. The incidence has been reported to be under 2% out of all endometrial cancers [13]. Given the lack of appropriate diagnostic criteria of this tumor, confusion with grade 3 endometrial carcinoma, high-grade sarcoma, carcinosarcoma, and adenosquamous carcinoma has been reported many times. In addition, nonkeratinizing squamous cell carcinoma and/or poorly differentiated adenocarcinoma of the uterine cervix may be misdiagnosed as undifferentiated carcinoma of the endometrium.

Undifferentiated carcinoma of the uterus usually shows aggressive behavior, and the disease progresses rapidly. In most cases, it is diagnosed at an advance stage. In this case, the disease had already spread throughout the patient's entire body at the time of diagnosis. It is conceivable that cancer developed during pregnancy or otherwise before and that cancer progression during pregnancy caused the premature delivery at 29 weeks. Pregnancy may have masked the insidious progression of this cancer.

Endometrial carcinomas are rare during adolescence. Those cases that do form are mostly estrogen dependent, and they show relatively good differentiation of cancer cells and have better prognoses. Pregnancy and the hormonal environments it creates are believed to exert negative effects on the development and growth of the endometrial carcinoma. In this case, however, cancer cells expressed neither ER nor PR, so the pregnancy-induced hormonal environment did not affect them. Instead, increased blood flow into the reproductive tract may have favored the progression of this cancer.

Shiomi et al. summarized 25 cases of pregnancy-associated endometrial carcinoma from the previous works going back to 1995 and their own case, of which grade 1 and grade 2 endometrial carcinoma accounted for 23 cases [14]. Vaccarello et al. have summarized 27 cases reported in 1997, and earlier, of which grade 1 endometrial carcinoma accounted for 25 cases [3]. These previous works indicate that endometrial carcinoma discovered during pregnancy or the postpartum period is mostly low-grade cancer and that favorable prognoses can be obtained. Out of all 52 patients in these two works, only two died of the reported cancer.

Kodama et al. reported a case with poorly differentiated adenosquamous carcinoma detected at 7 months postpartum [6]. In this case, cancer cells showed prominent ability to invade the myometrium and remarkable metastatic potential surrounding lymph nodes rather than tumor formation in the endometrial cavity. Our case showed similar invasive activity into the myometrium and other parts of the reproductive tract. However, we find it extraordinary that cancer was detected at only 2 months postpartum and that pregnancy was terminated at 29 weeks due to spontaneous labor. These facts suggest that the premature delivery was caused by cancer in this case. As pregnancy progresses, the amniotic cavity expands with time and with fetal growth. In such an environment, it is likely that undifferentiated carcinoma cells derived from the endometrium could invade deeper tissues rather than forming a tumor mass.

In the present case, the origin of the cancer remains uncertain. However, the clinical features of the cancer and the immunohistochemical profiles of the cancer cells strongly suggest an endometrial origin.

## Figures and Tables

**Table 1:** Immunohistochemical expression of the tumor cells.

BerEP4	4+
CEA	4+
p16	4+
Vimentin	4+
TTF-1	3+
CDX2	3+
CK7	2+
CK20	−
CK34*β*E12	2+
CK5/6	+
EMA	+
p63	−
GATA3	−
GCDFP15	−
CA125	−
ER	−
PgR	−
AR	−
HER2/neu	−
CD56	−
Synaptophysin	2+
Chromogranin	−
ALK	−
ALK (FISH)	−
EGFR (FISH)	−

## Data Availability

The data that support the findings of this study are available from the corresponding author upon reasonable request.
